# Imaging the breast following neoadjuvant chemotherapy: does improvement of imaging findings in the axilla predict pathological response in the breast?

**DOI:** 10.1186/bcr3285

**Published:** 2012-11-09

**Authors:** A Williams, KJ Litton, A Borley, P Young

**Affiliations:** 1 School of Medicine, Cardiff University, Cardiff, UK; 2 Cardiff and Vale Breast Centre, University Hospital Llandough, Cardiff, UK; 3 Velindre Hospital, Velindre NHS Trust, Cardiff, UK

## Introduction

Neoadjuvant chemotherapy is an effective treatment in patients with locally advanced breast cancer. It is being used more frequently and imaging is used to monitor disease response. Imaging response in the axilla was correlated with the final breast histology.

## Methods

Patients who had undergone primary chemotherapy, pre and post chemotherapy MRI and surgery from 2009 to present were included. The MRI and histology reports were reviewed.

## Results

Of 20 patients, 18 had abnormal nodes on the first MRI; of these nine had a complete imaging response and five had axillary disease on final pathology. Of the nine that had abnormal nodes on their final MRI, all but one had metastatic disease on final pathology. Of the nine patients with a response on axillary imaging, two had a complete pathological response in the breast. The other had an average tumour size of 53 mm (range 16 to 120 mm). Of the patients who did not respond radiologically in the axilla, three had a complete pathological response in the breast and the remainder had an average tumour size of 44 mm (range 1.7 to 90 mm).

## Conclusion

Although MRI is used to predict response to chemotherapy it may underestimate and overestimate disease burden. A radiological response in the axilla does not predict a response in the breast and the two areas need to be assessed independently of each other. Surgical planning must take into consideration the pre-chemotherapy disease status in each area.

